# Interrogating and Quantifying In Vitro Cancer Drug Pharmacodynamics via Agent-Based and Bayesian Monte Carlo Modelling

**DOI:** 10.3390/pharmaceutics14040749

**Published:** 2022-03-30

**Authors:** Marios Demetriades, Marko Zivanovic, Myrianthi Hadjicharalambous, Eleftherios Ioannou, Biljana Ljujic, Ksenija Vucicevic, Zeljko Ivosevic, Aleksandar Dagovic, Nevena Milivojevic, Odysseas Kokkinos, Roman Bauer, Vasileios Vavourakis

**Affiliations:** 1 Department of Mechanical and Manufacturing Engineering, University of Cyprus, Nicosia 2109, Cyprus; demetriades.marios@ucy.ac.cy (M.D.); hadjicharalambous.myrianthi@ucy.ac.cy (M.H.); ioannou.eleftherios@ucy.ac.cy (E.I.); okokki@mail.ntua.gr (O.K.); 2 Department of Science, Institute for Information Technologies Kragujevac, University of Kragujevac, 34000 Kragujevac, Serbia; marko@kg.ac.rs (M.Z.); nevena.milivojevic@uni.kg.ac.rs (N.M.); 3 Faculty of Medical Sciences, Human Genetics, University of Kragujevac, 34000 Kragujevac, Serbia; biljana.ljujic@medf.kg.ac.rs (B.L.); zeljko.ivosevic@medf.kg.ac.rs (Z.I.); 4 Department for Pharmaceutical Technologies, Faculty of Medical Sciences, University of Kragujevac, 34000 Kragujevac, Serbia; vucicevic.ksenija@medf.kg.ac.rs; 5 Oncology and Radiotherapy Centre, Faculty of Medical Sciences, University of Kragujevac, 34000 Kragujevac, Serbia; dagovic@sbb.rs; 6 Department of Computer Science, University of Surrey, Guilford GU2 7XH, UK; 7 Department of Medical Physics and Biomedical Engineering, University College London, London WC1E 6BT, UK

**Keywords:** mathematical oncology, in silico, simulation, Variational Bayesian Monte Carlo, cytostatics, drug testing

## Abstract

The effectiveness of chemotherapy in cancer cell regression is often limited by drug resistance, toxicity, and neoplasia heterogeneity. However, due to the significant complexities entailed by the many cancer growth processes, predicting the impact of interference and symmetry-breaking mechanisms is a difficult problem. To quantify and understand more about cancer drug pharmacodynamics, we combine in vitro with in silico cancer models. The anti-proliferative action of selected cytostatics is interrogated on human colorectal and breast adenocarcinoma cells, while an agent-based computational model is employed to reproduce experiments and shed light on the main therapeutic mechanisms of each chemotherapeutic agent. Multiple drug administration scenarios on each cancer cell line are simulated by varying the drug concentration, while a Bayesian-based method for model parameter optimisation is employed. Our proposed procedure of combining in vitro cancer drug screening with an in silico agent-based model successfully reproduces the impact of chemotherapeutic drugs in cancer growth behaviour, while the mechanisms of action of each drug are characterised through model-derived probabilities of cell apoptosis and division. We suggest that our approach could form the basis for the prospective generation of experimentally-derived and model-optimised pharmacological variables towards personalised cancer therapy.

## 1. Introduction

Clinical development of anti-cancer drugs is a resource-intensive and time-consuming process, which requires effective novel preclinical platforms for screening compounds targeting neoplasia [1]. The therapeutic success of anti-cancer drugs is highly dependent upon numerous factors including the type of cancer, the timing, and dosage of administered drugs, as well as combinations of these. In vitro tumour models are a valuable tool in the pursuit of exploring substances that can exhibit anti-cancer activity, as well as for assessing their effectiveness [2]. Cancer-mimicking in vitro models offer a reductionist approach to facilitate a detailed primary screening of anti-cancer drugs, thus preventing drugs with insufficient anti-tumour activity from entering preclinical animal testing. Cancer biologists and pharmaceutics experts rely on well established in vitro models, such as two-dimensional or three-dimensional tumouroid models, microfluidic devices and bioreactors, etc. Further to in vitro testing, pharmacological testing using in vivo (animal) models is carried out to assess bio-availability, toxicity, and therapeutic efficacy of candidate compounds [3]. Thus, the choice of an appropriate in vitro and in vivo tumour model at the stage of testing can facilitate timely screening, pre-clinical or clinical, of cytotoxic anti-cancer agents as it can enable reduction in both financial and time costs [4]. However, drug screening is a complicated and interdisciplinary process, which involves a laborious path that starts from the identification of competent drug targets (i.e., enzymes, receptors, ion channels), target validation through biochemical assays, assay development to screen modulators and high-throughput screening of a large number of chemical substances against biological targets (tumour cells). Furthermore, molecular descriptors can be optimised to improve selectivity and drug potential of lead candidate compounds for drug screening and drug development—a process known as lead optimisation.

**Figure 1:**
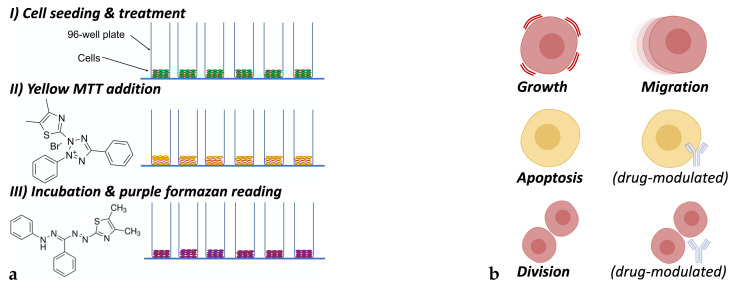
(**a**) Graphical representation of the MTT viability assay procedure: from cell seeding and treatment to reduction of yellow MTT to purple formazan crystals for tracking of the metabolic activity of viable cells to estimate the amount of cells survived after drug treatment; (**b**) agent-based model mechanisms accounted for in cell (agent) behaviour include cell growth, migration, division, and apoptosis. The latter two are also modulated by the presence of cytostatics.

In addition to the experimental methods, theoretical approaches have experienced an uptake in medicine, as they help simplify and conceptualise underlying biological complexities. Indeed, concepts such as symmetry and symmetry-breaking are employed also to formalise cancer dynamics [5,6]. Notably, theoretical models cover topics in oncology across a wide range of scales and complexities, from genetic and individual-cell processes to population-level incidence. Mathematical models in cancer biomedicine can be broadly classified with respect to the type of physics and level of biology they encompass, as well as the involved spatio-temporal scales of the (cancer) system that is under consideration, as illustrated in Figure 1 of the Yankeelov et al. review [7]. Along those lines, the advent of computing and the significant advances in high-performance computing algorithms have rendered in silico modelling increasingly more practical and popular in cancer biology, precision medicine and personalised therapy [8,9]. This is strongly motivated by the rising number of available pre-clinical models (extending from in vitro to in vivo) and clinical data, which require suitable robust computational methods for their analysis. As such, modelling the complex biology of cancer and predicting how tumours would respond to therapies requires in silico approaches that can handle various types of data-derived information and combine diverse theoretical methods on multiple temporal and spatial scales [10,11].

At the tissue or organ scale, physics-based in silico models often employ continuum-based (fluid, solid) biomechanics governed by partial differential equations. Such models have been reported to simulate avascular or/and vascularised tumour growth, biofluid flow and drug transport phenomena. Relevant to the above, pharmacokinetics/pharmacodynamics-based models have been able to simulate the exchange of drugs among compartments and accumulation of drugs within a particular organ or the cancerous tissue [12].

Another category of in silico models in biomedicine that has been receiving increased popularity is that of spatially discrete, particle-based models which describe entities as agents, hence referred to as agent-based models [13]. The major types of these models include (a) lattice-based models (e.g., cellular automata, lattice gas cellular automata, Potts models), (b) off-lattice models (e.g., vertex and center-based models, deformable cell models) and (c) and hybrid continuum–discrete models, e.g., see [14,15,16,17]. Agent-based modelling (ABM) is a computational approach where elements of a system are modelled as discrete and autonomous entities that can interact with one another, as well as with their local spatial environment. Agents typically express behaviours that are governed by internal rules that are inheritable [18]. Within the cancer context, agents can represent individual cells that can have different phenotypic behaviour (e.g., neoplastic or healthy, proliferative or necrotic, etc.), while they can also respond to external stimuli, for instance mechanical interactions, (intra-/transcellular) signalling, and other important factors occurring during the evolution of cancer. It is beyond the scope of this paper, however, to provide an exhaustive survey about ABM in cancer, and so we refer the reader to the following reviews: [7,19].

In this manuscript, we propose a combined in vitro/in silico approach to evaluate and characterise the anti-tumoural activity of commonly used chemotherapeutic agents. The in vitro experiments we conducted report on the effects of selected cytostatics on two established cancer cell lines (colorectal carcinoma and breast adenocarcinoma) through pertinent cytotoxicity laboratory tests. The in silico modelling framework we developed combines off-lattice agent-based modelling and a Variational Bayesian Monte Carlo method to simulate cancer cell culture development, with or without the presence of a cytotoxic agent. The in vitro evidence is used within the computer-based modelling procedure for estimating the probabilities of cell division and apoptosis for each treatment scenario, thus providing insights into the action of each chemotherapeutic drug. Our work contributes to cancer research by reporting a new combined in vitro/in silico methodological approach for cancer drug screening through the quantification of cytotoxicity effects on cancer cell proliferation and apoptosis. The paper starts with the methodological description of the in vitro experiments and cytotoxicity analysis, and continues with the presentation of the in silico framework, including ABM and model parameter optimisation. Subsequently, we report on the experimental results of the cytotoxicity laboratory tests, the computational model assessment and verification, and the corresponding model-derived evaluation of the effect of the different treatment regimes. Important findings of this work are summarised in Section 4, along with the main limitations of this work, while future prospects in cancer pharmacodynamics are discussed.

## 2. Methods

### 2.1. In Vitro Procedure

#### 2.1.1. In Vitro Experiments of Cell Culturing

We examined cytotoxic and thus anti-proliferative activity of selected cytostatics on two model systems: a human colorectal carcinoma HCT-116 cell line and a human breast cancer MDA-MB-231 cell line. The cell lines of low passages were acquired from the European Collection of Authenticated Cell Cultures and were maintained in Dulbecco’s Modified Eagle Medium (DMEM) (D5796; Sigma-Aldrich Chemical Company, St. Louis, MO, USA) supplemented with 10% foetal bovine serum (F4135-500ML; Sigma-Aldrich Chemical Company, St. Louis, MO, USA) and 1% penicillin/streptomycin (P4333-100ML; Sigma-Aldrich Chemical Company, St. Louis, MO, USA) in 75 cm^2^ culture flasks. The cells were grown in an incubator in a humidified atmosphere with 5% CO_2_ at a physiological temperature of 37 °C, and, after a few passages and a confluence of about 80%, the cells were analysed with the MTT assay. Figure 1a depicts in a graphical manner the in vitro cytotoxicity testing procedure, which starts off with the cancer cell seeding and subsequent exposure to the cytotoxic drug (treatment). Then, as described in the following paragraph, the yellow MTT substance is reduced to purple formazan where subsequent absorbance reading on 550 nm provides indication about the cancer cell viability due to treatment.

#### 2.1.2. Cytotoxicity Assay

The capability of tested cytostatics to reduce the cell growth of two different cell lines was assessed by a standardised MTT assay (Laboratory for Bioengineering protocol CB-005). An approximate 10,000 cell population per well (in 96-well microplates) were seeded and cultured in an incubator for 24 h to allow cell adhesion. After incubation, the cells were treated with cytostatics—the cytostatics used in this study are commonly used in clinical practice. For the colon cancer treatment, 5-Fluorouracil, Oxaliplatin, Leucovorin, and Irinotecan were used, while, for the breast cancer treatment, Endoxan, Paclitaxel, Docetaxel, and Doxorubicin were used. The cytostatics/antibodies were kindly donated by the University Clinical Centre of Kragujevac, Serbia. The chosen concentration range was standardised from 0.1 to 500 μM, while all drugs were dissolved in DMEM. The cytotoxic effect on cancer cells was measured 24 and 72 h after treatment by estimation of the number of cells that survived at that stage of the experiment. The MTT assay was based on spectrophotometric measurement of reduction of 3-(4,5-dimethylthiazol-2-yl)-2,5-diphenyltetrazolium bromide (MTT, 158990010; Acros Organics, New Jersey, USA) to purple formazan crystals subsequently dissolved in dimethyl sulfoxide (DMSO) (D/4121/PB15; Fisher Chemical, Fair Lawn, NJ, USA). The absorbance measurements at 550 nm were performed using a Rayto 2100C microplate reader. The cytotoxicity was quantified by the ratio of the absorbance of the treated cells divided by the absorbance of the negative control (untreated cells) and multiplied by 100 to obtain the percentage of the viable cells [20].

#### 2.1.3. Statistical Analyses

Cytotoxic activity was experimentally interrogated in six repetitions for each treatment dose scenario. Statistical analyses were evaluated by using the one-way ANOVA test for multiple comparisons by employing the SPSS statistical software package (SPSS for Windows, version 17, 2008). The *IC*_50_ values were calculated from the dose curves using the CalcuSyn software. These values represent the amount of the substance required in order to decrease the number of cells by 50%. In principle, an anti-cancer drug is anticipated to express a clear selectivity towards cancer cells. Thus, the in vitro screening permitted us to measure drug- and cancer (cell line) specific effects—the experimental measurements yielded *IC*_50_ value estimates, expressed in molar concentrations.

### 2.2. In Silico Procedure

#### 2.2.1. Probabilistic Agent-Based Model

Our in silico approach employs a probabilistic agent-based model (ABM) designed to recapitulate our in vitro experiments and help understand the dynamics of cancer cell responses to treatment. In principle, ABM is a complex-system modelling method that assumes autonomous and interactive ‘agents’. Each agent is denoted a particle positioned in space following an off-lattice modelling approach. This work assumed a two-dimensional space, albeit ABM can generalise in three dimensions seamlessly. Thus, agents were set to freely move in a disk plane (diameter 2.4 mm) under specific restrictions for agent–agent overlap and collapse, while we assumed that the boundary of the simulation domain extends periodically to encompass the entire in vitro well. The behaviour of each agent was determined by simple rules and interactions with other agents, as well as external stimuli: the cytotoxic drugs in this work. These individual rules and interactions created patterns, structures, and produced time-varying outcomes for the entire biological system, which was not explicitly programmed into the model.

In this study, agents represented individual cells that were considered as being either of the human colorectal carcinoma HCT-116 or of the human breast cancer MDA-MB-231 cell line. Figure 1b depicts the cell mechanisms considered in the ABM formulation of our in silico model. Agents, for both cancer cell lines, were modelled to (i) grow and mature, (ii) migrate following random movement, (iii) divide symmetrically, and (iv) commit to apoptosis. In this study, the latter two mechanisms of cell behaviour were allowed to be modulated in the presence of a cytotoxic agent, thus defining the mode of action of the drugs under consideration. Contrary to this, cell growth and migration were not affected by the action of any chemotherapeutic agents. Drug presence was included in the model as a chemical substance whose concentration was homogeneous everywhere in the extracellular space of the simulation domain (disk plane of the well). The concentration of the drug was set constant in time and was not affected by the cells’ uptake of the drug. In addition, drug transport inside the well was almost instantaneous (drug convection or diffusion was neglected) while zero drug dissipation was not accounted for in the extracellular matrix. The series of rules set to describe all agents’ (cells) behaviour is outlined in Algorithm 1 box, where *P*_•_ denotes the probability of occurrence for a cell biological process. Presently, the probability for cell migration and cell growth was set fixed to 99% and 90%, respectively, whereas the probability for cell apoptosis and cell division (either programmable or induced by the drug) were the subject of investigation in the parametric analysis that follows in the Section 3. Regarding cell migration, their movement was taken as a combination of random walk movement on a fixed average rate of 0.2 μm per minute, and a chemotactically-driven cell migration towards higher O_2_ saturation level on a fixed average rate of 0.1 μm per minute. Cells were modelled to interact mechanically with one another when in close proximity (enough to warrant contact or overlap) by applying Newton (repellent) forces. In addition, matrix–cell interaction forces were neglected in this work, since cells in the in vitro tests were set in a free suspension medium, and cell adhesion is ignored.
**Algorithm 1** Cell behaviour algorithm that is iterated for all cells (agents) and for all time-steps of an ABM simulation.**if** random_number $\leq P_{\mathrm{apoptose}}$ **then**      Remove cell from simulation      **return****if** random_number $\leq P_{\mathrm{migrate}}$ **then**      Displace cell**if** random_number $\leq P_{\mathrm{grow}}$ **then**      Develop cell diameter, *d*, unless $>d_{\max}$      **return****if** random_number $\leq P_{\mathrm{divide}}$ **then**      Create new copy of (mother) cell: (daughter) cell      Half the volume of (mother & daughter) cells

#### 2.2.2. Model Parameter Optimisation

The unknown parameters of our ABM simulations were estimated using the Variational Bayesian Monte Carlo (VBMC) method [21]. VBMC belongs to the family of Bayesian inference methods, which can provide a posterior distribution of the unknown parameters while accounting for model error and parameter uncertainty. VBMC is a suitable Bayesian inference framework when dealing with expensive black-box likelihoods. It combines variational inference with Gaussian-process based, active-sampling Bayesian quadrature providing an approximate posterior and lower bound to the model evidence. Unlike the Markov-chain Monte Carlo and other known methods, VBMC can perform efficiently in realistic scenarios with complex likelihood functions and higher dimensions (up to D=10). VBMC is designed to alleviate the computational burden associated with the multiple model evaluations of such methods by introducing computationally inexpensive statistical surrogates (e.g., Gaussian processes) to approximate the posterior parameter distribution. Additionally, VMBC uses the uncertainty in the statistical surrogate to guide active sampling, which enables further enhancements in the computational efficiency of the approach [21].

The VBMC framework has been employed within this work to efficiently estimate approximate posterior distributions and lower bounds to the model evidence of our models. Specifically, the two unknown ABM parameters for each treatment scenario were estimated numerically, namely the probability of cell apoptosis, *P*_apoptose_, and the probability of cell division, *P*_divide_. Gaussian distributions were used as priors for both parameters, while the prior means were set to μ(*P*_apoptose_) = 0.2 and μ(*P*_divide_) = 0.15, respectively. Parameters were estimated by fitting the in silico model-derived cell viability to the relevant in vitro values.

#### 2.2.3. Computer Software Implementation

The agent-based modelling method for this work was implemented in a *C++* project invitro_neuro that was built and maintained on Bitbucket. A snapshot of the simulator can be downloaded from Figshare. Link to access the computer code from Figshare: https://doi.org/10.6084/m9.figshare.19387094.v1. It employed at its core the open-source software platform BioDynaMo (version 1.1.70). BioDynaMo [16] was used as it is designed to meet the computational demands in our ABM by employing highly optimised code that leverages high-performance computing simulations. Furthermore, implementation of the VBMC used was based on the open-source GitHub project vbmc that is coded in MATLAB. All in silico experiments were performed on a MacBook Pro (macOS Catalina 10.15.7) with a 2.3 GHz 8-Core Intel Core i9, and 64 GB (2667 MHz DDR4) memory. Each individual ABM simulation run (using our in-house project invitro_neuro was executed using two threads in a Bourn-again shell) required approximately 2—3 s, whereas each batch of a full optimisation algorithm run (using the VBMC algorithm implementation in MATLAB) required 342.1 ± 36.6 s.

## 3. Results

### 3.1. In Vitro Experiments

**Figure 2:**
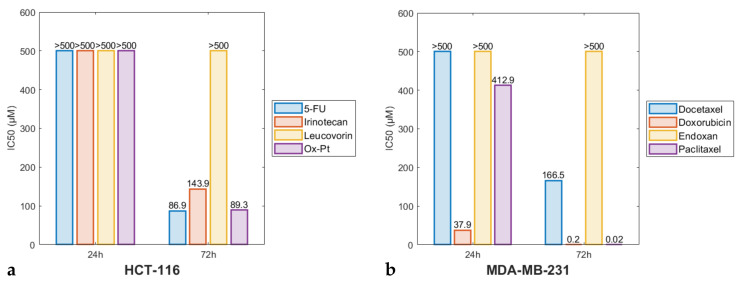
The toxicity effects against cells (*IC*_50_ values) of tested cytostatics on (**a**) HCT-116 and (**b**) MDA-MB-231 cells after 24 and 72 h of exposure to each drug (see figure legend).

Cytotoxicity in vitro experiments were performed to investigate the biological influence of selected commercial cytostatics against colorectal carcinoma HCT-116 and breast adenocarcinoma MDA-MB-231 cell lines. Figure 2 presents the cytotoxic effect (*IC*_50_ values) of the cytostatics tested on each cancer cell line respectively after 24 and 72 h of exposure to the drug.

**Figure 3:**
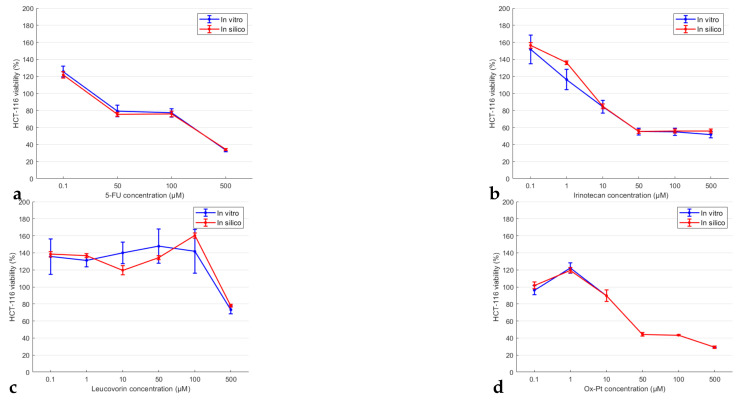
Comparison plots between the in vitro (blue lines) and the in silico (red lines) results after 72 h of HCT-116 cells’ incubation with respect to the drug type and concentration used per treatment scenario, drug: (**a**) 5-Fluorouracil; (**b**) Irinotecan; (**c**) Leucovorin; and (**d**) Oxaliplatin (Ox-Pt).

**Figure 4:**
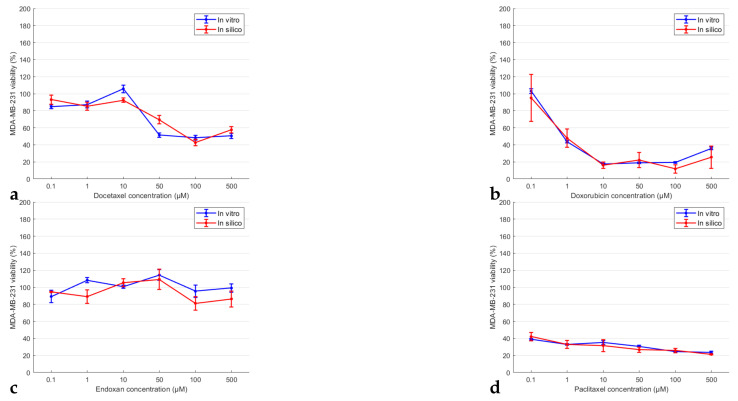
Comparison plots between the in vitro (blue lines) and the in silico (red lines) results after 72 h of MDA-MB-231 cells’ incubation with respect to the drug type and concentration used per treatment scenario, drug: (**a**) Docetaxel; (**b**) Doxorubicin; (**c**) Endoxan; and (**d**) Paclitaxel.

In Figure 3 and Figure 4, the viability of HCT-116 and MDA-MB-231 cell lines is plotted out respectively (blue line and symbol) for each single-treatment scenario with respect to the relevant cytostatic concentration, following 72 h of incubation. Line plots for both cell lines are also provided, following 24 and 72 h of incubation, in Figures S1 and S2 respectively of the Supplementary Material document.

The in vitro treatment of HCT-116 cells revealed the most prominent cytotoxic character of 5-Fluorouracil (5-FU), Oxaliplatin (Ox-Pt), and Irinotecan after 72 h, while Leucovorin exerted a marginal cytotoxic effect at low to moderate drug concentrations (see also Figure S1). Besides Leucovorin, the rest of the three drugs show time and dose dependent lowering of the cell viability. Calculation of the *IC*_50_ values (Figure 2a) revealed that 5-FU, Ox-Pt, and Irinotecan exerted significant effects 72 h after treatment (*IC*_50_^5-FU^ = 86.9 μM, *IC*_50_^Ox-Pt^ = 89.3 μM, *IC*_50_^Irinotecan^ = 143.9 μM). In MDA-MB-231 cells treatment, Doxorubicin exerted predominant anticancer activity in both treatment periods (see also results depicted in Figure S2). A significant effect after 72 h was also exerted by Paclitaxel and Docetaxel, while Endoxan did not show significant cytotoxic effects (see Figure 4). *IC*_50_ values presented in Figure 2b show the same trend for these drugs. Thus, Doxorubicin and Paclitaxel with a *IC*_50_ < 50 μM can be considered as a very aggressive treatment option against the above-mentioned breast cancer cell line.

### 3.2. In Silico Model Verification

Next, the in silico modelling procedure was tested and verified against the in vitro data of the cytotoxicity assay experiments. Figure 3 shows the in silico predicted HCT-116 cell line viability 72 h following incubation, when different cytotoxics were administered at different concentration values. Similarly, in Figure 4, in silico predicted MDA-MB-231 cell line viability is compared against the in vitro cell viability. Graphical representations of the development of the cell populations in 2D are shown in Figures S3 and S4, at three time points (1 h, 24 h, and 72 h of incubation) and for different drug concentrations. Animations to the aforementioned figures can be accessed through the following project on Figshare: https://figshare.com/projects/In_vitro_cancer_drug_pharmacodynamics_via_Agent-based_and_Bayesian_Monte_Carlo_modelling/133802 (accessed on 20 February 2022).

Comparing the in vitro, blue lines, to the in silico results, red lines, the model demonstrates its capacity to capture the experimentally measured cell viability very well. Indeed, the in vitro viability (for both cell lines and at most cytostatics) demonstrated a decline with increased drug concentration, see Figure 3a. This trend is also evident in the in silico simulation outcomes. In rare cases, the model fails to match exactly the in vitro results, particularly when significant fluctuations are present, as can be seen in Figure 4a.

The model accuracy was also quantified by assessing the correlation of the in vitro and in silico cell viability through a linear regression model. Model accuracy was assessed by calculating the $R^{2}$, the standard error of the coefficient estimate, and the corresponding *p*-value. The values to these quantities are listed in Table 1 (see also Figure S5) and were calculated by taking the cell viability results from Figure 3 and Figure 4. Examples of the linear regression fits for both cell lines and for the different chemotherapeutic agents are provided in Figures S6 and S7 respectively.

To assess the accuracy and reliability of the in silico model, we quantified the impact of selected model parameters on the final number of cancer cells (i.e., 72 h after incubation) by considering the untreated case scenario (control) for both cell lines. Parameter sweeps were performed for assessing the behaviour of model parameters, whereby the apoptosis and division probability parameters of the agent-based model, *P*_apoptose_ and *P*_divide_, were varied by ±10% compared to their control values. The control values for both probabilities were estimated through the VBMC method described in Section 2.2.2. However, the baseline parameter values for the HCT-116 control were *P*_apoptose_ = 17.5% and *P*_divide_ = 15.65%, while, for the MDA-MB-231 control, the parameter values were *P*_apoptose_ = 19.34% and *P*_divide_ = 17.36%.

**Figure 5:**
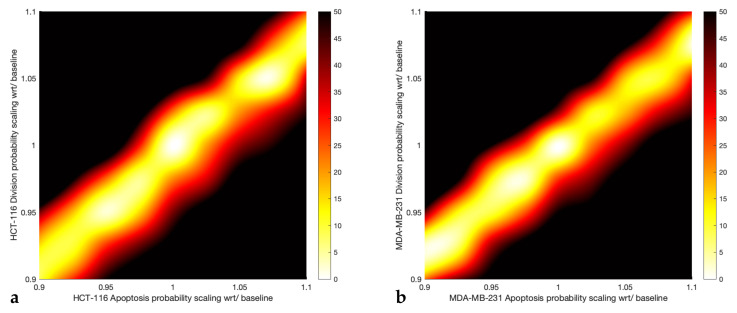
Heat-maps of the absolute error of the simulation results for (**a**) the HCT-116 and (**b**) MDA-MB-231 cell line, respectively, under control conditions. Horizontal and vertical axes describe the scaling of *P*_apoptose_ and *P*_divide_, respectively. The colour-bar represents the percentage of absolute error with black areas corresponding to ≥50%.

Figure 5 presents heat-maps of the absolute error of the cell population number (HCT-116 and MDA-MB-231 cell lines, respectively) under control conditions. The horizontal and vertical axes indicate the scaling of *P*_apoptose_ and *P*_divide_, respectively. A scaling factor of 1 for the apoptosis and division probabilities corresponds to the baseline pair of parameter values adopted in the control simulations, as demonstrated in the verification tests in Figure 3 and Figure 4. The diagonal band in Figure 5 indicates the area where absolute errors are low (<±15%), i.e., the cell viability predictions are similar amongst scenarios within the band. In other words, limited changes to both *P*_apoptose_ and *P*_divide_ produce relatively small changes to the simulation results. Therefore, the pattern of the absolute error distribution (shown as a diagonal band) in Figure 5 suggests that the two parameters are strongly coupled, as pairs of parameters that differ significantly can produce very similar results; this is depicted as multiple minima of the absolute error in the contour plot of Figure 5. Intuitively, the coupling can be justified by the fact that cell viability declines following the administration of a chemotherapeutic drug could result by either reducing the probability of division or by increasing the probability of apoptosis. In turn, the lack of a unique minimum of the absolute error implies that it is not possible to pinpoint a unique value for both the probability of division and the probability of apoptosis. However, it is important to highlight that there is a limit to the change of the parameters that can be performed over parameter sweeps. As illustrated in Figure S8, more severe changes to apoptosis or division probabilities result in rapid changes in the number of cancer cells predicted through the ABM. This is because the model is designed (see Algorithm 1) to check first for cell (agent) apoptosis and then (if viable) for division, as the main goal is to measure the toxicity of the drugs. Consequently, considerable values for *P*_apoptose_ lead towards the cell population decline—if a cell dies, then it cannot contribute to the overall cell growth regardless to its propensity to divide and create new cancer cell copies.

### 3.3. Quantification of Drug Effects in Cell Response

Following the in silico model verification tests, we employed the experimental data of the cytotoxic agents for both in vitro model systems (human colorectal carcinoma HCT-116, human breast cancer MDA-MB-231) within our modelling framework to further assess drug cytotoxicity effects. The mechanisms of action of each drug were examined through the model-predicted probabilities of cancer cell division, *P*_divide_, and apoptosis, *P*_apoptose_. In order to circumvent any biases due to differences between the various treatment experiments, the probabilities of division and apoptosis were normalised with respect to their corresponding control values for each drug screening experiment: P^*^_divide_ = *P*_divide_(Treated) /*P*_divide_(Control), and P^*^_apoptose_ = *P*_apoptose_(Treated) / *P*_apoptose_(Control). Original, non-normalised values for the apoptosis and division probabilities are provided in Figure S9 for the HCT-116 cell line and in Figure S10 for the MDA-MB-231 cell line. In addition, Tables S1 and S2 summarise the apoptosis and division probability parameter values for each simulation scenario for both cell lines, respectively.

Due to the coupling between the probabilities of division and apoptosis (see Figure 5), a new index was introduced to allow us to reliably characterise the effect of each treatment on the two cell lines. In particular, although the absolute values of the probabilities cannot be retrieved, their ratio can be reliably estimated. Accordingly, further insights into each chemotherapeutic agent were sought by considering an index of cancer survival, namely the cancer cell survival probability ratio, which is defined as the ratio of the normalised division and apoptosis probabilities: P^*^_divide_ / P^*^_apoptose_. In principle, values of the survival probability ratio close to 1 could either signify no significant deviations from the control values or correspond to similar changes in the probabilities of division and apoptosis—the latter scenario being less probable as a drug would likely act by increasing apoptosis and decreasing division and vice versa. On the other hand, values of cancer cell survival below 1 denote a stronger anti-tumoural effect.

**Figure 6:**
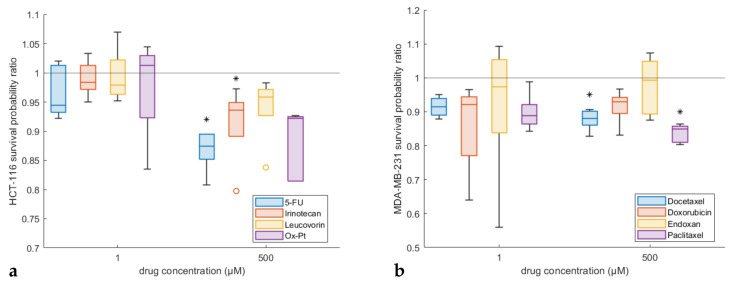
Box plots of the simulation results of the cancer cell survival probability ratio (normalised division probability to normalised apoptosis probability fraction. Plots for (**a**) HCT-116 and (**b**) MDA-MB-231 cell lines, respectively, for every treatment scenario at two extreme drug concentrations. Each scenario is illustrated in a different colour; HCT-116: blue for 5-FU, red for Irinotecan, yellow for Leucovorin, and purple for Ox-Pt drug; MDA-MB-231: blue for Docetaxel, red for Doxorubicin, yellow for Endoxan, and purple for Paclitaxel drug. *t*-tests at a 5% significance level are denoted with a “*” above corresponding boxes.

Figure 6a,b report using box plots for the survival probability ratio for the HCT-116 and MDA-MB-231 cancer cells, respectively, for low (1 μM) and high (500 μM) concentrations. The survival probability ratio can be thought of as a complementary index to cell viability in Figure 3 and Figure 4, as it can also characterise the mechanisms of action of chemotherapeutic agents, i.e., whether they act through reducing cell division or by inducing apoptosis. Complementary to Figure 6, Figure S11 shows the box plots of the reciprocal quantity to the cancer cell survival probability ratio defined above. Statistical analysis was subsequently performed to quantify the differences in the drug’s effect at low and high concentrations. An unpaired *t*-test with unequal variance was employed to test the hypothesis that the mean of cancer cell survival for the low-concentration drug is lower than the mean of the high-concentration drug. Statistically significant results (*p*-value ≤ 0.05) are depicted with asterisks in Figure 6. In addition, Tables S3 and S4 tabulate the mean and standard error of the survival percentage, as quantified through the in silico experiments, for the HCT-116 and MDA-MB-231 cell lines, respectively.

In view of the in silico HCT-116 results demonstrated in Figure 6a, we can conclude that the cancer cell survival was near 1 for all four drugs for low concentrations, which suggest that all cytostatics did not have a strong anti-tumoural effect at such concentration levels. This fact is in agreement with the experimental and simulation results shown in Figure 3, whereby cell viability was over 100% at low concentrations (1 μM) for all drugs. In contrast, for higher concentrations, the cancer cell survival fell consistently below 1 in Figure 6a, suggesting a stronger cytotoxic effect for all drugs at 500 μM. Furthermore, the predicted cell survival values for 5-FU and Ox-Pt were lower than those of Irinotecan and Leucovorin, in agreement with Figure 3, where the cell viability of 5-FU and Ox-Pt was approximately 35%, while the viability for Irinotecan and Leucovorin was roughly 60% and 80%, respectively. Notably, the statistical tests indicated that the cancer cell survival was significantly lower for higher concentrations compared to lower concentrations, for 5-FU and Irinotecan. This finding indicates that these drugs could mainly act by inducing apoptotic effects rather than by suppressing division of the cancer cells.

The in silico MDA-MB-231 results in Figure 6 show that the median cancer cell survival is lower than 1 for Docetaxel, Doxorubicin, and Paclitaxel for low concentrations, as shown in Figure 4a,b,d. This means that—unlike the corresponding HCT-116 cell survival results—the above drugs have an apoptotic effect on breast cancer cells even at low concentration levels. Endoxan gives for MDA-MB-231 cell survival a value close to 1—implying a negligible effect on the cell behaviour—which agrees with Figure 4c, where the cell viability is higher than 100% for 1 μM. The cancer cell survival index remains high for higher concentrations of Endoxan, suggesting that the cancer cell survival is not affected by the concentration increase (in agreement with the in vitro results shown in Figure 4c). Similarly, Doxorubicin, which exhibited very strong anti-tumoural effects even at low concentrations in Figure 2, did not present substantial variation in the survival of the cancer cells under investigation. Interestingly, both Docetaxel and Paclitaxel caused a statistically significant decrease in cancer cell survival at higher concentrations, which suggests that their dominant mechanism of action is to induce cell apoptosis.

## 4. Discussion

Computational models are increasingly involved in biomedicine and pharmaceutical development. One of the main reasons for this is that in silico methods offer a complementary approach to conduct research and development, in addition to wet-lab experimental work and purely analytical techniques. Indeed, such a precision medicine approach offers numerous benefits such as lower costs, reduced need for animal experiments, or the potential for highly adaptive and individualised patient care. In particular, it constitutes an attractive method to deal with complex symmetry-breaking processes, which play a crucial role during cancer progression [5,22]. We thus demonstrate in this contribution a proof-of-concept that combines in silico, using agent-based modelling (ABM), with pertinent in vitro data to provide highly relevant information and guidance on the usage of drugs for cancer treatment.

Our computational results complement our experimental cancer model results. In particular, we find that well-established chemotherapeutic drugs have a differential impact on fundamental tumourigenesis parameters—in particular, the ratios between proliferative and apoptotic activities. We suggest that this ratio can become a quantitative measure of cancer progression and viability with a prognostic potential. For instance, drugs could be chosen in a context-specific manner, taking into account indicators of the proliferative rate of a patient’s cancer vs. the propensity to migrate and metastasise. Our computational results also indicate that the effects of these drugs significantly differ with regard to the concentrations at which they should be administered. Despite the fact that this study focused on a specific drug mode of action (i.e., suppression of cancer cell division and enhancement of apoptosis potential), the proposed in silico ABM methodology can be readily extended to account for other combinations of cytotoxic effects, e.g., reduce cell invasiveness, down-regulate differentiation to metastatic phenotypic behaviours, etc. However, quantitative measures of these aspects can be inferred by combining ABMs with wet-lab experiments, accelerating hypotheses’ generation and testing. From a purely experimental perspective, the treatment of colorectal and breast carcinoma cells revealed that not all cytostatics exert strong anticancer activity, even though these drugs are commonly used in clinical practice. This is not surprising because the protocols for patient treatment include the use of combinations of these drugs depending on the type and stage of the cancer. For example, in the treatment of colon cancer, the *Folfox* protocol represents treatment with Ox-Pt, Leucovorin, and 5-FU on the first and second day. This combination administered on these two days represents Cycle 1. The next cycle is applied after 14 days. The number of cycles depends on clinical evaluation. Another example is protocol *Folfiri*, which represents a combination of Irinotecan, Leucovorin and 5-FU in the very similar fashion as in the *Folfox* protocol. The synergism of applied drugs is crucially important for patient outcome. Similar to colon cancer, breast cancer patients also undergo protocol-based therapy. An *AC* protocol represents a combination of Doxorubicin with Endoxan administered once every three weeks. Paclitaxel and Docetaxel are used as single therapeutics administered in weekly intervals prescribed by the medical expert. We are currently conducting in vitro experiments of combinations of chemotherapeutic drugs, with preliminary results indicating synergistic drug effects in cancer regression. As such, we plan to carry out a large-scale parametric analysis study, using our in silico method, to interrogate and optimise combinatory chemotherapeutic protocols.

Notably, our approach has demonstrated in this paper a moderate computational footprint, which offers the prospect of exploring a broad model parameter space (e.g., cell migration, cell differentiation, and invasiveness). Therefore, it further supports the usage of ABM for determining the most effective treatment schedules or strategies. For instance, the in silico can be used to generate causal predictions on the efficacy of different treatment durations, or scheduling protocols that are not present in the initial training data.

Equally important to incorporating in vitro experimental data is the capability to take into account other background knowledge on cellular metabolism and drug interactions that can be seamlessly incorporated into our mechanistic model. This distinguishes our approach from other computational techniques, such as deep neural networks, where statistical models are generated from the training dataset. Such ‘black box’ approaches do not mechanistically model biophysical processes, and so it is not possible to include previously established rules, or extrapolate scenarios that are not implicit in the training data. In contrast, our approach allows for generating verifiable hypotheses also for treatment scenarios not captured by the experimental data.

Last but not least, a particularly promising avenue is to employ our approach to interrogate and test candidate drugs and tailored combinations of drugs that will likely have the most beneficial impact in killing specific cancers. In this regard, they constitute a complementary approach that can be combined with promising and innovative laboratory methods such as lab-on-a-chip technologies, which allow for incorporating interactions with the tumour microenvironment. Along those lines, the integration of patient-specific information, relying on genetic and imaging data, could enable a computationally assisted and personalised treatment approach in oncology.

We note that this work constitutes a proof-of-concept for the proposed (combined agent-based and Bayesian Monte Carlo) modelling approach of in vitro chemotherapeutic experimental results. Prospectively, further experimental validation based on measurements obtained from other research labs are required to solidify the biomedical suitability and clinical applicability of our in silico approach. We suggest the development of studies where computational modelling is employed to infer experimentally verifiable predictions, and the validation of these. In this way, contradictory hypotheses can be reliably discarded, and the models that have the highest explanatory power can be identified. We highlight that our data are freely accessible and invite interested researchers to conduct additional, independent validation of our results. Along those lines, the incorporation of additional model components and experimental data are necessary to realise the usage of our modelling approach in preclinical studies. Indeed, recent experimental models allow for obtaining relevant data with regard to interactions with immune cells [23] and the tumour microenvironment [24,25]. We see significant opportunities in leveraging such methods in combination with computational modelling to employ precision medicine in oncology.

Overall, we demonstrate the applicability of ABM as a powerful complementary tool to experimental methods for probing chemotherapeutic impact. In the future, we anticipate increased uptake of in silico methods for modelling cancer growth and the impact of treatments, in particular mechanistic computational models as well as statistics based models. This is because such models have the capability to generate concrete and verifiable predictions even when using model parameters that are not directly inferred from experimental data. The mechanistic modelling approach presented in this paper, including physics-based and pharmacokinetics/pharmacodynamics models, can help inform about likely cancer evolution and prognosis.

## Figures and Tables

**Table 1:** $R^{2}$ values, standard error of the estimate (SEE), and *p*-values for all drugs and for both cancer cell lines.

Cytotoxic Agent	$R^{2}$	SEE	*p*-Value
5-FU	0.9990	4.084	0.000499
Irinotecan	0.9789	10.3902	0.000168
Leucovorin	0.753259089	15.8834	0.025021
Ox-Pt	0.994785615	3.0653	0.00001
Docetaxel	0.788004721	12.8863	0.01821
Doxorubicin	0.965406075	8.0124	0.000454
Endoxan	0.24742795	14.1856	0.315406
Paclitaxel	0.847478031	3.424	0.00921

## Data Availability

All data of this study are contained within the article, the Supplementary Material document, and the Figshare project: https://figshare.com/projects/In_vitro_cancer_drug_pharmacodynamics_via_Agent-based_and_Bayesian_Monte_Carlo_modelling/133802 (accessed on 20 February 2022).

## Supplementary Materials

The following supporting information can be downloaded at: https://www.mdpi.com/article/10.3390/pharmaceutics14040749/s1, Figure S1: Line plots of the in vitro results for the HCT-116 cell line expressed with respect to the drug concentration for every treatment scenario, Figure S2: Line plots of the in vitro results for the MDA-MB-231 cell line expressed with respect to the drug concentration for every treatment scenario, Figure S3: Snapshots of the in silico HCT-116 cell population development for the treatment scenario using Irinotecan, Figure S4: Snapshots of the in silico MDA-MB-231 cell population development for the treatment scenario using Paclitaxel, Figure S5: Bar charts of the standard error of the estimate evaluated after linear regression of the in silico results against the in vitro, Figure S6: Line plots of the linear regression for HCT-116 cell line viability for the various drug treatment scenarios, Figure S7: Line plots of the linear regression for MDA-MB-231 cell line viability for the various drug treatment scenarios, Figure S8: Heat-maps of the absolute error of the simulation results for HCT-116 and MDA-MB-231 under control conditions, Figure S9: Box plots of the in silico model results for the HCT-116 cell line, Figure S10: Box plots of the in silico model results for the MDA-MB-231 cell line, Figure S11: Box plots of the simulation results of the normalised apoptosis probability ratio to the corresponding normalised division probability ratio for HCT-116 and MDA-MB-231, Table S1: HCT-116 cell line apoptosis and division probabilities as predicted in silico, Table S2: MDA-MB-231 cell line apoptosis and division probabilities as predicted in silico, Table S3: HCT-116 cell line survival percentage as quantified in silico, Table S4: MDA-MB-231 cell line survival percentage as quantified in silico.
