# Supplementary material for: Interrogating and Quantifying In Vitro Cancer Drug Pharmacodynamics via Agent-Based and Bayesian Monte Carlo Modelling
Source: Pharmaceutics. 2022 Mar 30;14(4):749. doi: 10.3390/pharmaceutics14040749 (PMC9029523; doi:10.3390/pharmaceutics14040749)
Supplement: Supplementary file 1 [file pharmaceutics-14-00749-s001.zip › pharmaceutics-1627816-supplementary.pdf]

Interrogating and Quantifying In Vitro Cancer Drug Pharmacodynamics via Agent-Based and Bayesian Monte Carlo Modelling

—

*Supplementary Material*

M. Demetriades, M. Zivanovic, M. Hadjicharalambous, E. Ioannou,
B. Ljujic, K. Vucicevic, Z. Ivosevic, A. Dagovic, N. Milivojevic, O. Kokkinos,
R. Bauer and V. Vavourakis

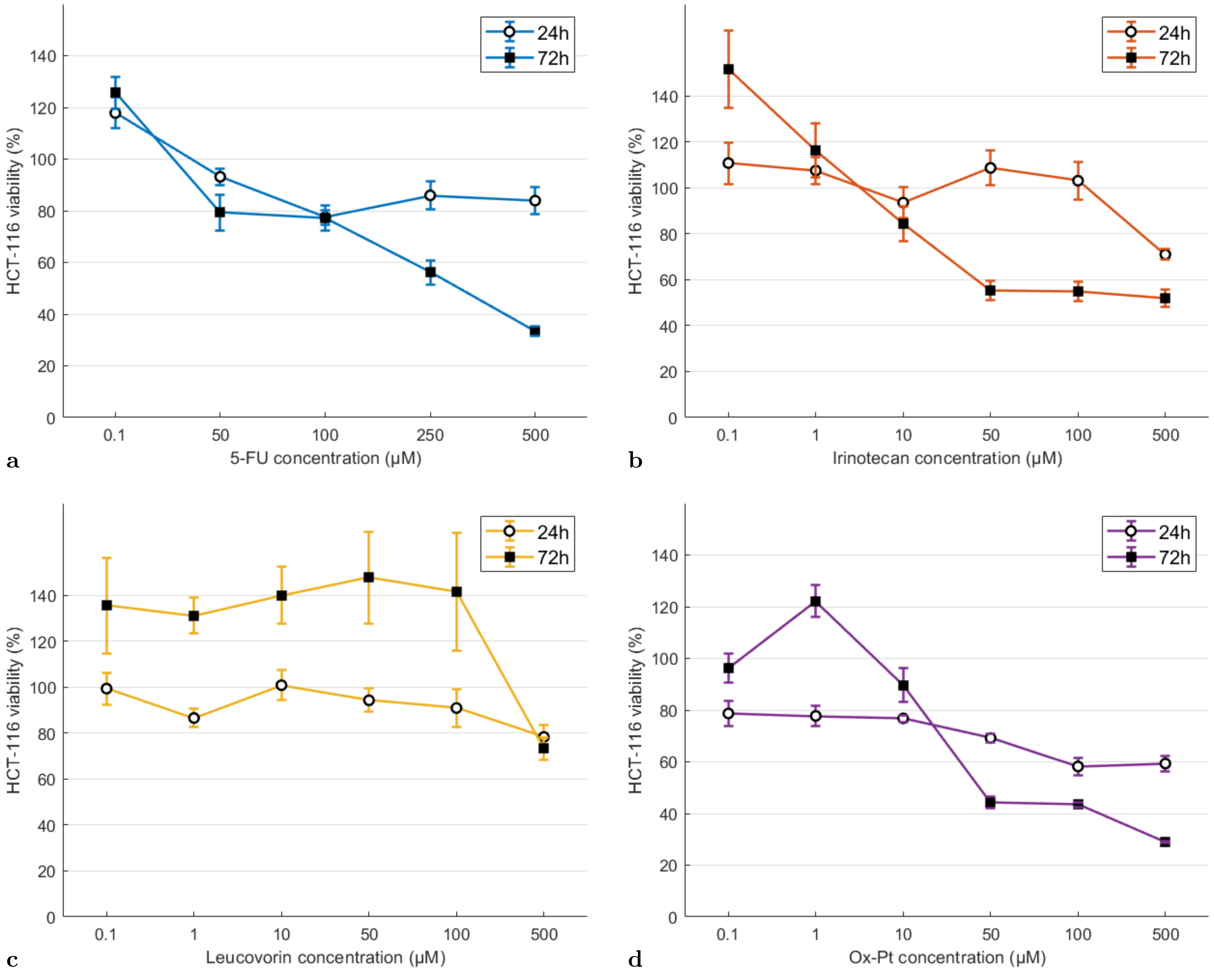


**Figure S1**: Line plots of the *in vitro* results for the HCT-116 cell line expressed with respect to the drug concentration for every treatment scenario: (**a**) 5-Fluorouracil (5-FU), (**b**) Irinotecan, (**c**) Leucovorin, and (**d**) Oxaliplatin (Ox-Pt) drug. Note, the horizontal axes are not in scale.

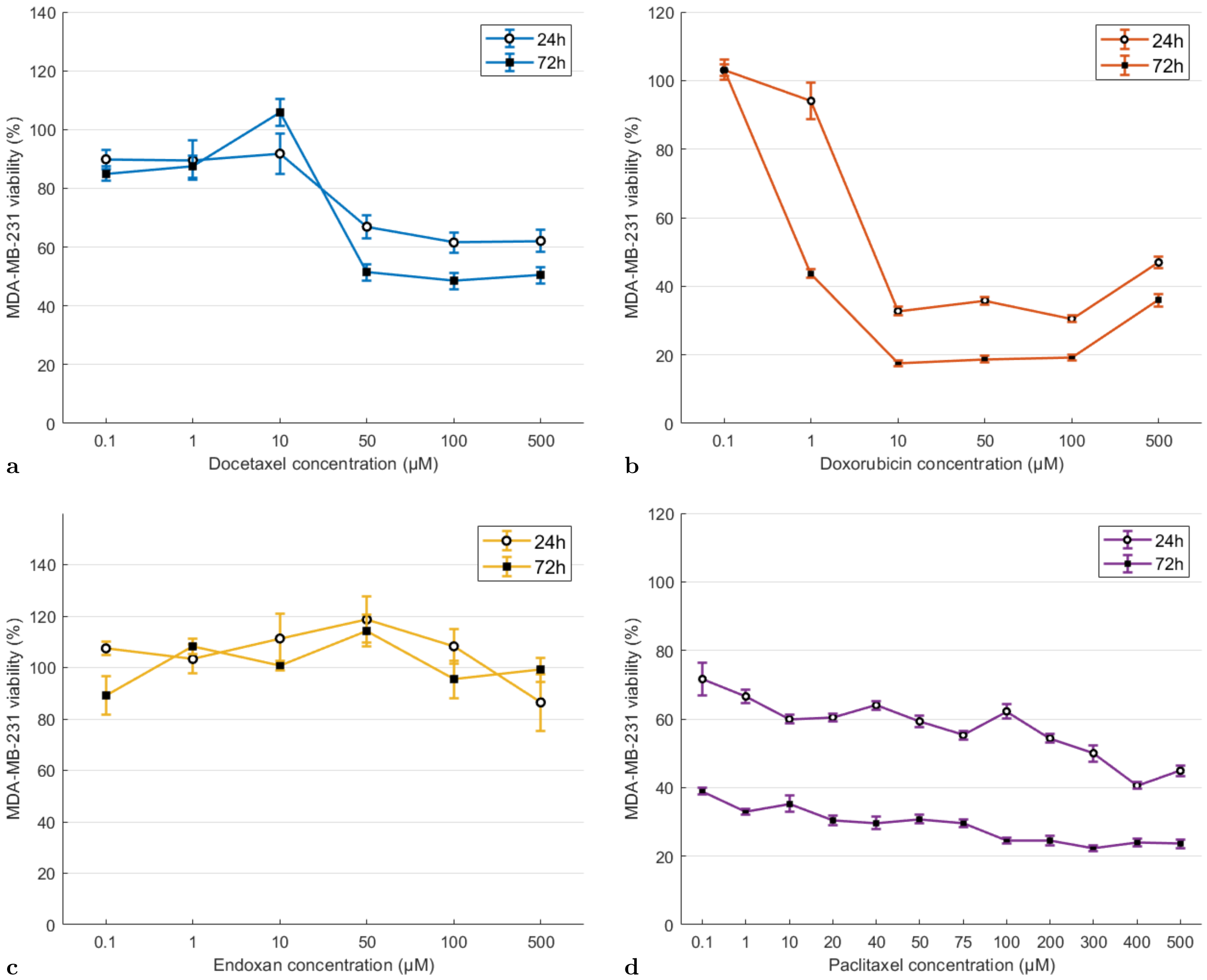


**Figure S2**: Line plots of the *in vitro* results for the MDA-MB-231 cell line expressed with respect to the drug concentration for every treatment scenario: (**a**) Docetaxel, (**b**) Doxorubicin, (**c**) Endoxan, and (**d**) Paclitaxel drug. Note, the horizontal axes are not in scale.

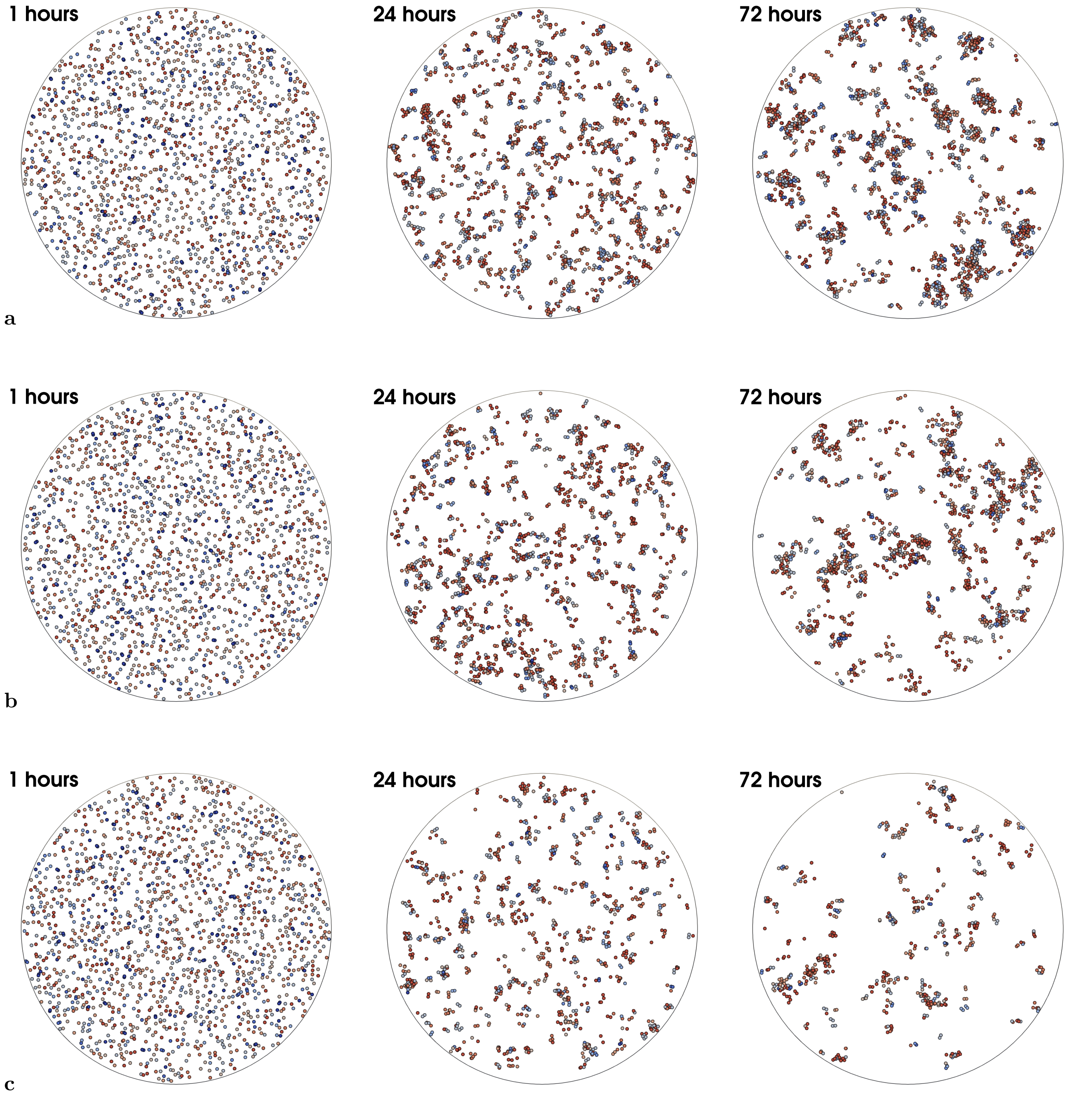


**Figure S3**: Snapshots of the *in silico* HCT-116 cell population development for the treatment scenario using Irinotecan at drug concentrations (**a**) 0 $\mu$M (Control), (**b**) 1 $\mu$M, and (**c**) 500 $\mu$M, the animations of which can be accessed in Figshare via DOI: 10.6084/m9.figshare.19258799.v1, DOI: 10.6084/m9.figshare.19258811.v1 and DOI: 10.6084/m9.figshare.19258820.v1 respectively.

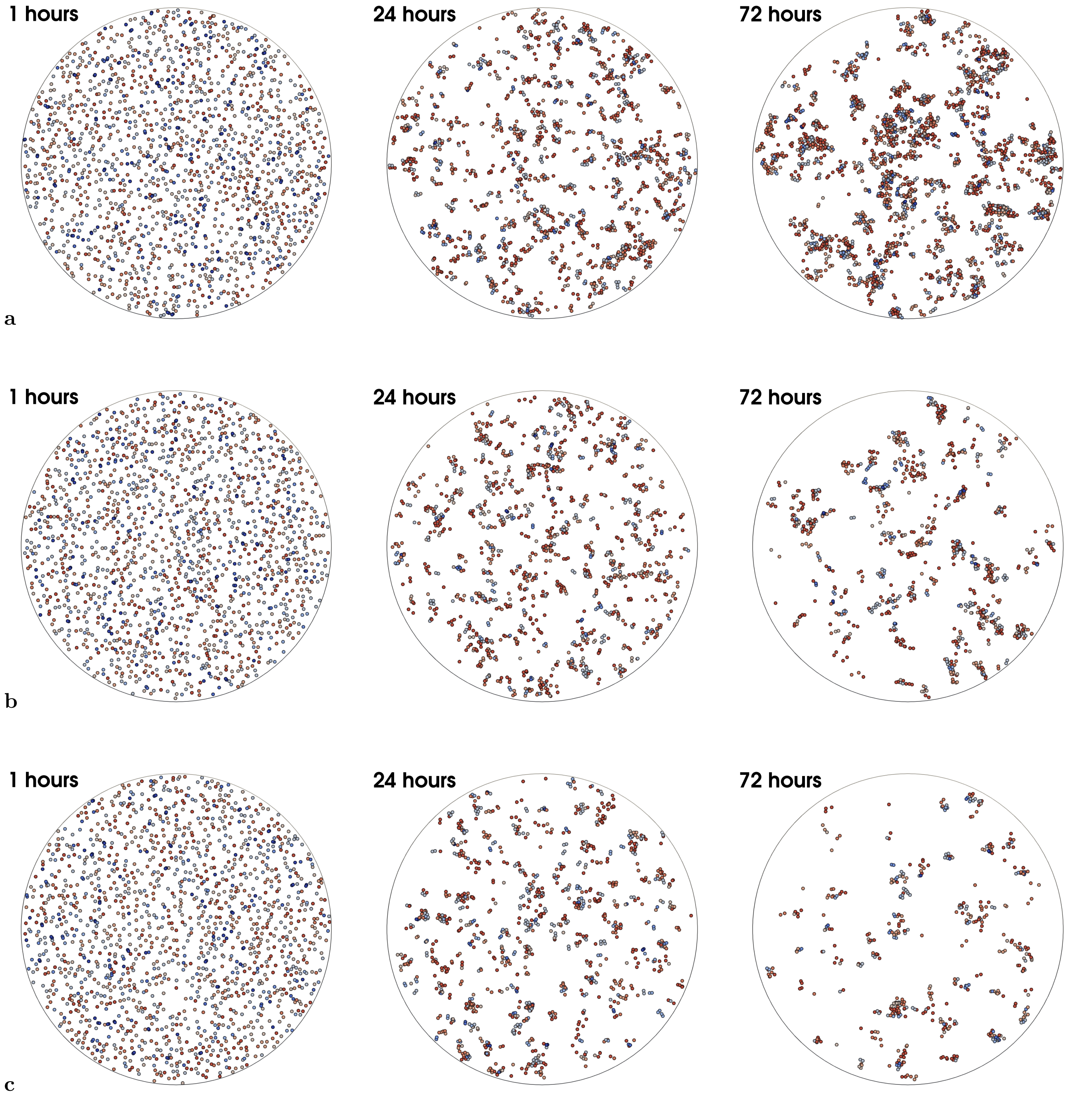


**Figure S4**: Snapshots of the *in silico* MDA-MB-231 cell population development for the treatment scenario using Paclitaxel at drug concentrations (**a**) 0 $\mu$M (Control), (**b**) 1 $\mu$M, and (**c**) 500 $\mu$M, the animations of which can be accessed in Figshare via DOI:10.6084/m9.figshare.19258826.v1, DOI:10.6084/m9.figshare.19258829.v1 and DOI:10.6084/m9.figshare.19258835.v1 respectively.

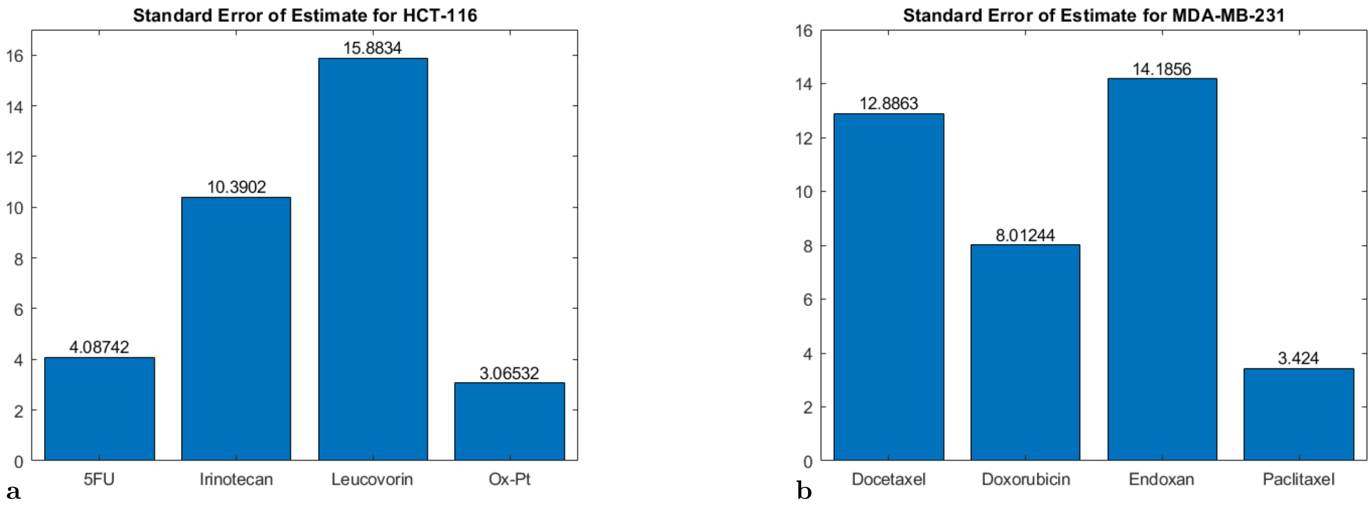


**Figure S5**: Bar charts of the standard error of the estimate evaluated after linear regression of the *in silico* results against the *in vitro* on the (**a**) HCT-116 and (**b**) MDA-MB-231 cell line respectively.

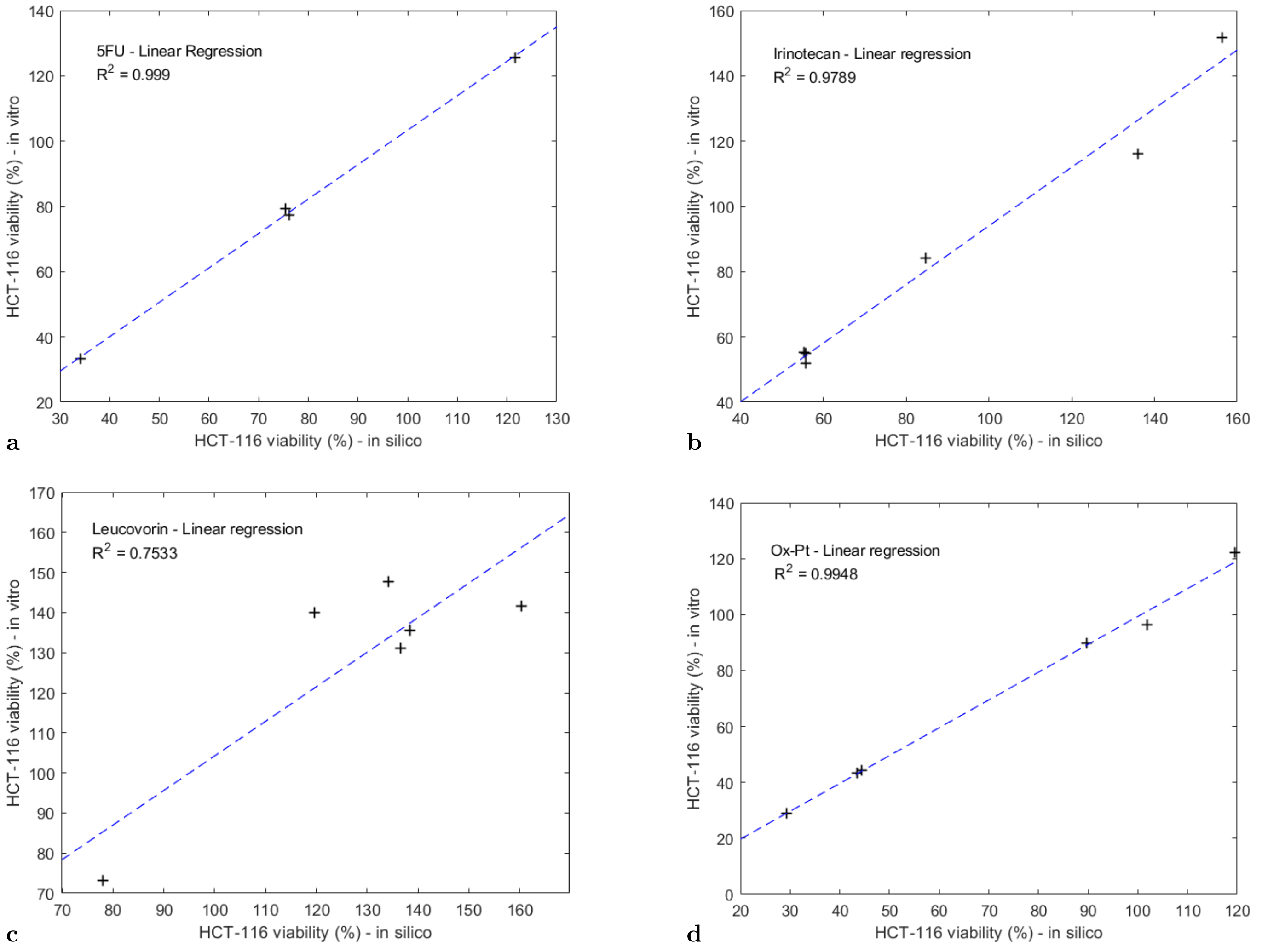


**Figure S6**: Line plots of the linear regression for HCT-116 cell line viability for the various drug treatment scenarios (*in vitro* versus *in silico* from Figure 3; $R^2$ values are provided at the legend of each plot).

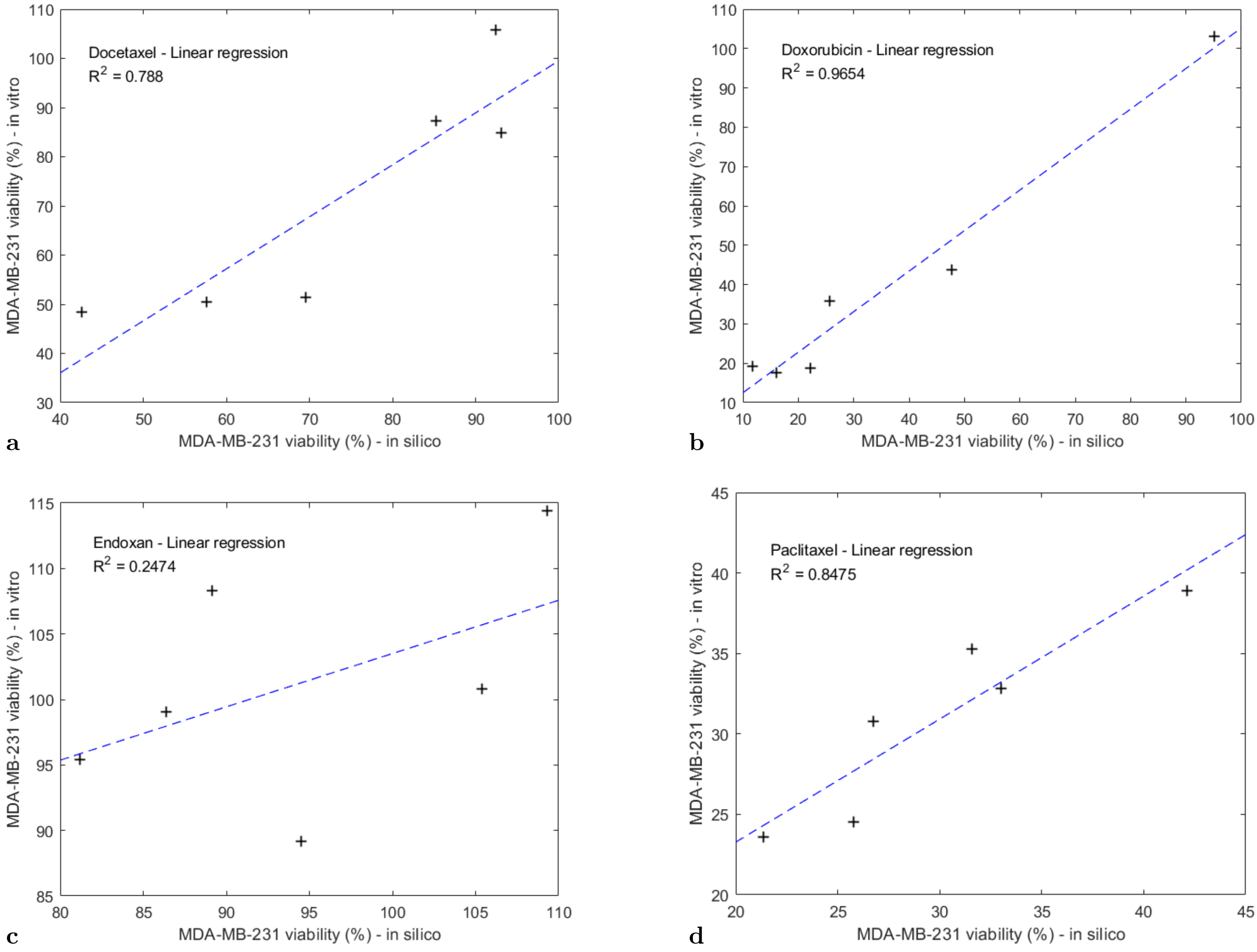


**Figure S7**: Line plots of the linear regression for MDA-MB-231 cell line viability for the various drug treatment scenarios (*in vitro* versus *in silico* from Figure 4; $R^2$ values are provided at the legend of each plot).

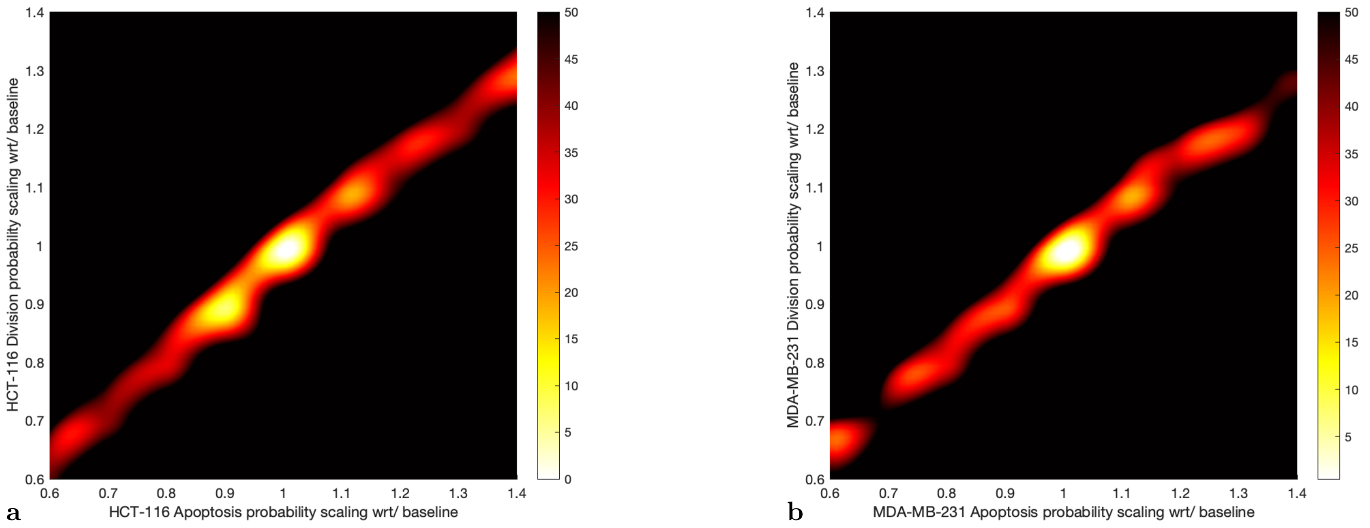


**Figure S8**: Heat-maps of the absolute error of the simulation results for (**a**) the HCT-116 and (**b**) MDA-MB-231 cell line respectively under control conditions. Horizontal and vertical axis describe the scaling of $P_{\text{apoptose}}$ and $P_{\text{divide}}$ respectively, with probability parameter values being scaled within a $\pm 40\%$ range with respect to the baseline. The colour-bar represents the percentage of absolute error with black areas corresponding to $\geq 50\%$.

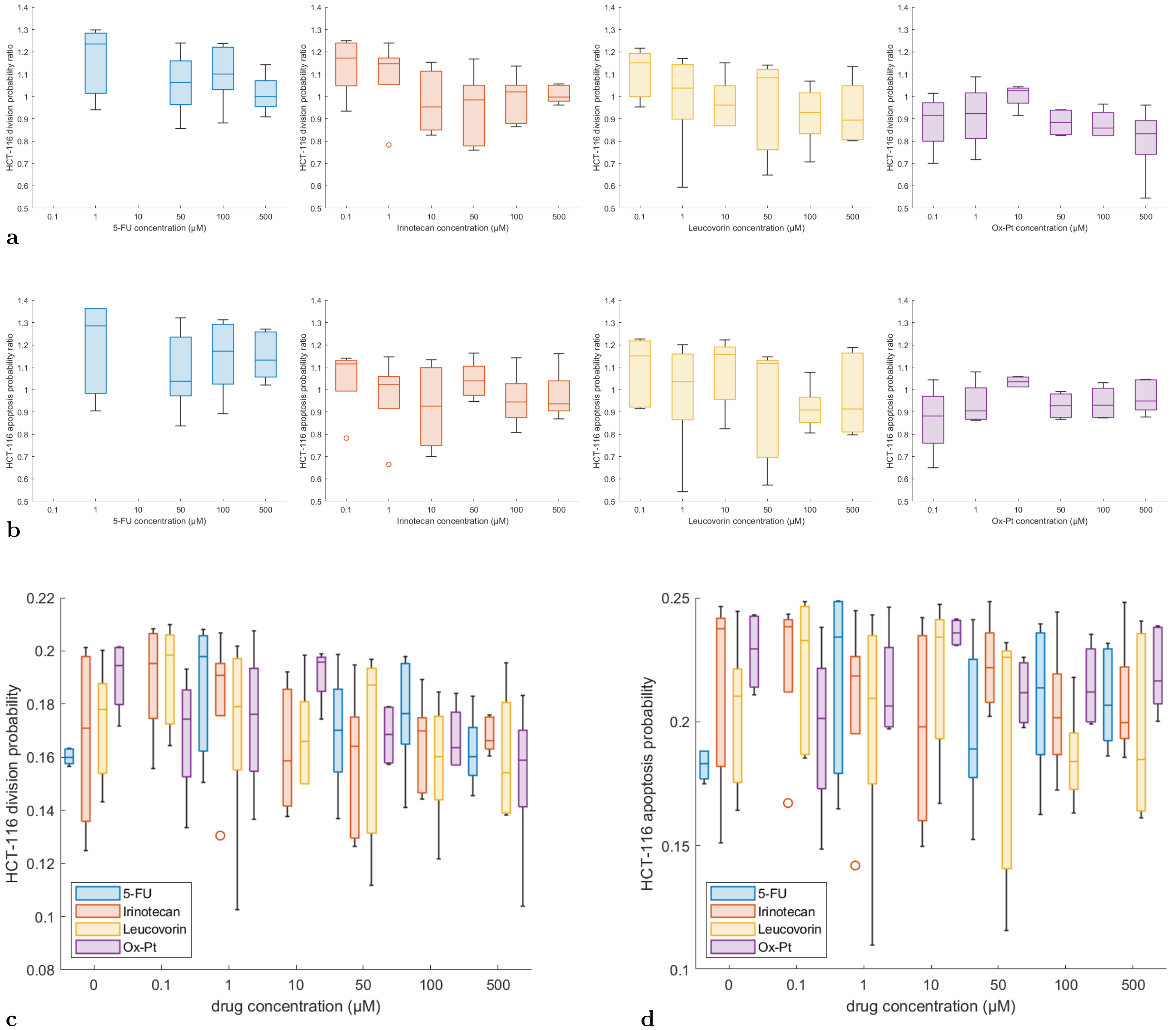


**Figure S9**: Box plots of the *in silico* model results for the HCT-116 cell line. (**a**) Division probability ratio and (**b**) apoptosis probability ratio simulation results with respect to the drug concentration for every treatment scenario (from left to right: 5-FU, Irinotecan, Leucovorin and Ox-Pt). The box plots of the above simulation results are aggregated with respect to the (**c**) division probability, and (**d**) apoptosis probability versus the drug concentration for every treatment scenario. Zero drug concentration corresponds to the untreated, control, case scenario while the numbers on the drug concentration axis are not in scale.

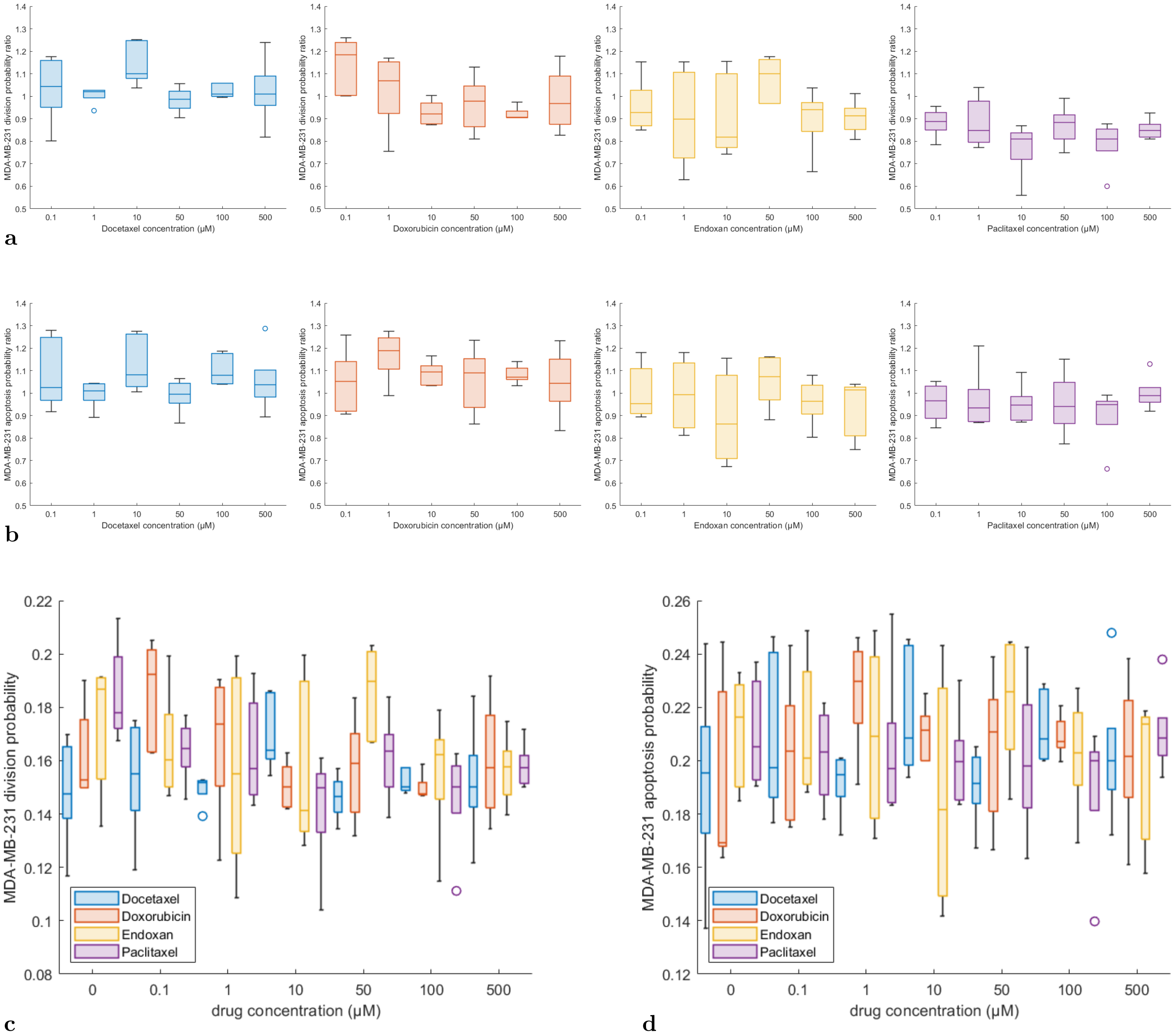


**Figure S10**: Box plots of the *in silico* model results for the MDA-MB-231 cell line. (**a**) Division probability ratio and (**b**) apoptosis probability ratio simulation results with respect to the drug concentration for every treatment scenario (from left to right: Docetaxel, Doxorubicin, Endoxan and Paclitaxel). The box plots of the above simulation results are aggregated with respect to the (**c**) division probability, and (**d**) apoptosis probability versus the drug concentration for every treatment scenario. Zero drug concentration corresponds to the untreated, control, case scenario while the numbers on the drug concentration axis are not in scale.

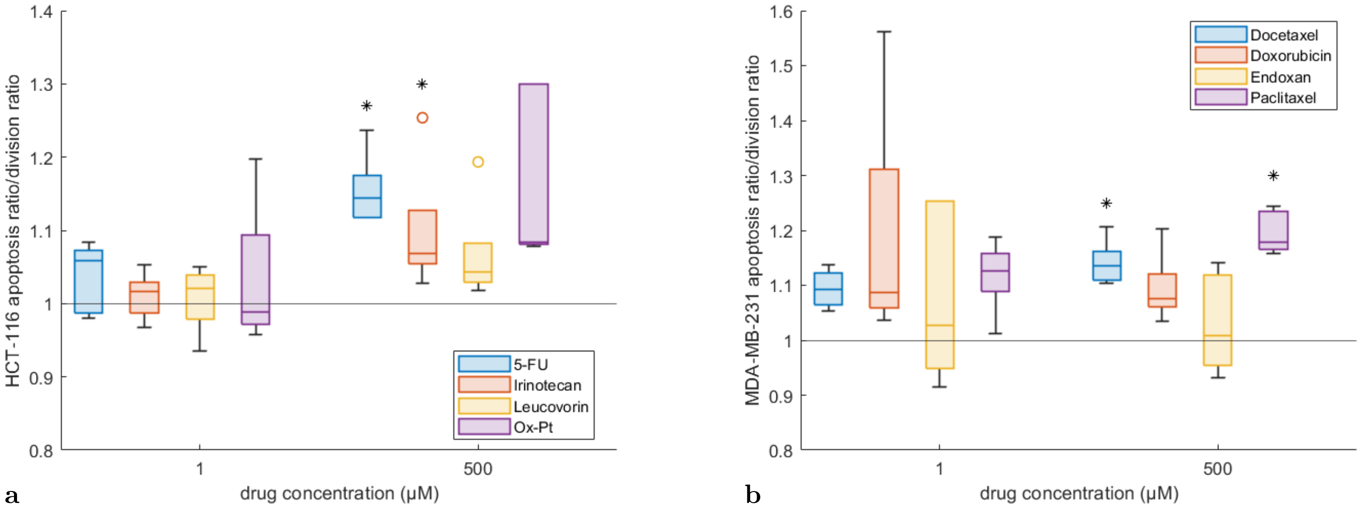


**a** **b**

**Figure S11**: Box plots of the simulation results of the normalised apoptosis probability ratio [$P_{\text{apoptose}}(\textsc{Treated})$ / $P_{\text{apoptose}}(\textsc{Control})$] to the corresponding normalised division probability ratio [$P_{\text{divide}}(\textsc{Treated})$ / $P_{\text{divide}}(\textsc{Control})$] for the (**a**) HCT-116 and (**b**) MDA-MB-231 cell line respectively. Plots include data for every treatment scenario at two extreme drug concentrations. Each scenario is illustrated in a different colour: blue for 5-FU, red for Irinotecan, yellow for Leucovorin, and purple for Ox-Pt drug tested on the HCT-116 cell line; blue for Docetaxel, red for Doxorubicin, yellow for Endoxan, and purple for Paclitaxel drug tested on the MDA-MB-231 cell line. T-tests at 5% significance level are designated with a "*" above corresponding boxes.

| **5FU** concentration ($\mu$M) | Apoptosis Probability | Division Probability |
|---|---|---|
| 0 (Control) | 0.18319 | 0.15984 |
| 0.1 | 0.18694 | 0.16618 |
| 50 | 0.22001 | 0.18116 |
| 100 | 0.16258 | 0.14104 |
| 500 | 0.1861 | 0.14545 |
| **Irinotecan** concentration ($\mu$M) | Apoptosis Probability | Division Probability |
| 0 (Control) | 0.24017 | 0.2 |
| 0.1 | 0.2406 | 0.20573 |
| 1 | 0.24473 | 0.20667 |
| 10 | 0.16341 | 0.14278 |
| 50 | 0.24852 | 0.19462 |
| 100 | 0.21099 | 0.17015 |
| 500 | 0.21099 | 0.17015 |
| **Leucovorin** concentration ($\mu$M) | Apoptosis Probability | Division Probability |
| 0 (Control) | 0.21024 | 0.178 |
| 0.1 | 0.18531 | 0.16448 |
| 1 | 0.19664 | 0.17264 |
| 10 | 0.1702 | 0.15008 |
| 50 | 0.11598 | 0.1118 |
| 100 | 0.16322 | 0.15121 |
| 500 | 0.1612 | 0.13821 |
| **Ox-Pt** concentration ($\mu$M) | Apoptosis Probability | Division Probability |
| 0 (Control) | 0.241664 | 0.20082 |
| 0.1 | 0.19773 | 0.1715 |
| 1 | 0.24625 | 0.20744 |
| 10 | 0.24147 | 0.19903 |
| 50 | 0.19776 | 0.1585 |
| 100 | 0.23531 | 0.18398 |
| 500 | 0.21637 | 0.16581 |

**Table S1**: HCT-116 cell line apoptosis and division probabilities as predicted *in silico* (see Figure 3).

| **Docetaxel** concentration ($\mu$M) | Apoptosis Probability | Division Probability |
|---|---|---|
| 0 (Control) | 0.24372 | 0.14535 |
| 0.1 | 0.17685 | 0.11921 |
| 1 | 0.1915 | 0.15267 |
| 10 | 0.19392 | 0.1543 |
| 50 | 0.16714 | 0.13441 |
| 100 | 0.2261 | 0.1575 |
| 500 | 0.19498 | 0.14958 |
| **Doxorubicin** concentration ($\mu$M) | Apoptosis Probability | Division Probability |
| 0 (Control) | 0.24432 | 0.17047 |
| 0.1 | 0.17513 | 0.16333 |
| 1 | 0.23892 | 0.19024 |
| 10 | 0.20002 | 0.15001 |
| 50 | 0.21077 | 0.16558 |
| 100 | 0.21077 | 0.19949 |
| 500 | 0.16098 | 0.13461 |
| **Endoxan** concentration ($\mu$M) | Apoptosis Probability | Division Probability |
| 0 (Control) | 0.191924 | 0.13556 |
| 0.1 | 0.20088 | 0.16991 |
| 1 | 0.24864 | 0.19925 |
| 10 | 0.14178 | 0.12821 |
| 50 | 0.24337 | 0.20023 |
| 100 | 0.16935 | 0.16225 |
| 500 | 0.21369 | 0.17468 |
| **Paclitaxel** concentration ($\mu$M) | Apoptosis Probability | Division Probability |
| 0 (Control) | 0.20536 | 0.17799 |
| 0.1 | 0.22154 | 0.17691 |
| 1 | 0.18324 | 0.14322 |
| 10 | 0.19993 | 0.15323 |
| 50 | 0.16321 | 0.13876 |
| 100 | 0.19539 | 0.15021 |
| 500 | 0.20849 | 0.15201 |

**Table S2**: MDA-MB-231 cell line apoptosis and division probabilities as predicted *in silico* (see Figure 4).

| **5-FU** concentration ($\mu$M) | mean (%) | std.(%) |
|---|---|---|
| 0.1 | 121.55 | 3.78 |
| 50 | 75.48 | 2.23 |
| 100 | 76.06 | 3.26 |
| 500 | 34.22 | 1.11 |
| **Irinotecan** concentration ($\mu$M) | mean (%) | std.(%) |
| 0.1 | 156.38 | 3.19 |
| 1 | 136.02 | 2.138 |
| 10 | 84.81 | 2.595 |
| 50 | 55.43 | 2.202 |
| 100 | 55.89 | 2.157 |
| 500 | 55.89 | 2.157 |
| **Leucovorin** concentration ($\mu$M) | mean (%) | std.(%) |
| 0.1 | 138.43 | 3.14 |
| 1 | 136.63 | 2.44 |
| 10 | 119.60 | 5.31 |
| 50 | 134.18 | 2.35 |
| 100 | 160.33 | 2.98 |
| 500 | 77.95 | 1.68 |
| **Ox-Pt** concentration ($\mu$M) | mean (%) | std.(%) |
| 0.1 | 101.80 | 3.96 |
| 1 | 119.62 | 3.13 |
| 10 | 89.67 | 6.63 |
| 50 | 44.33 | 2.09 |
| 100 | 43.41 | 0.84 |
| 500 | 29.21 | 1.27 |

**Table S3**: HCT-116 cell line survival percentage as quantified *in silico* (mean and standard error).

| **Docetaxel** concentration ($\mu$M) | mean (%) | std.(%) |
|---|---|---|
| 0.1 | 93.06 | 5.41 |
| 1 | 85.29 | 4.65 |
| 10 | 92.45 | 2.51 |
| 50 | 69.60 | 4.71 |
| 100 | 42.60 | 3.81 |
| 500 | 57.66 | 3.90 |
| **Doxorubicin** concentration ($\mu$M) | mean (%) | std.(%) |
| 0.1 | 95.08 | 27.68 |
| 1 | 47.77 | 10.72 |
| 10 | 16.10 | 3.57 |
| 50 | 22.16 | 8.76 |
| 100 | 11.78 | 4.93 |
| 500 | 25.61 | 13.10 |
| **Endoxan** concentration ($\mu$M) | mean (%) | std.(%) |
| 0.1 | 94.50 | 0.50 |
| 1 | 89.16 | 8.00 |
| 10 | 105.36 | 4.88 |
| 50 | 109.32 | 12.03 |
| 100 | 81.19 | 8.03 |
| 500 | 86.36 | 9.27 |
| **Paclitaxel** concentration ($\mu$M) | mean (%) | std.(%) |
| 0.1 | 42.11 | 4.84 |
| 1 | 33.01 | 4.72 |
| 10 | 31.58 | 6.83 |
| 50 | 26.73 | 3.14 |
| 100 | 25.76 | 2.45 |
| 500 | 21.34 | 0.48 |

**Table S4**: MDA-MB-231 cell line survival percentage as quantified *in silico* (mean and standard error).
